# Comprehensive reduction of amino acid set in a protein suggests the importance of prebiotic amino acids for stable proteins

**DOI:** 10.1038/s41598-018-19561-1

**Published:** 2018-01-19

**Authors:** Rei Shibue, Takahiro Sasamoto, Masami Shimada, Bowen Zhang, Akihiko Yamagishi, Satoshi Akanuma

**Affiliations:** 1 Faculty of Human Sciences, Waseda University, 2-579-15 Mikajima, Tokorozawa, Saitama 359-1192 Japan; 2 Department of Applied Life Science, Tokyo University of Pharmacy and Life Sciences, 1432-1 Horinouchi, Hachioji, Tokyo, 192-0392 Japan

## Abstract

Modern organisms commonly use the same set of 20 genetically coded amino acids for protein synthesis with very few exceptions. However, earlier protein synthesis was plausibly much simpler than modern one and utilized only a limited set of amino acids. Nevertheless, few experimental tests of this issue with arbitrarily chosen amino acid sets had been reported prior to this report. Herein we comprehensively and systematically reduced the size of the amino acid set constituting an ancestral nucleoside kinase that was reconstructed in our previous study. We eventually found that two convergent sequences, each comprised of a 13-amino acid alphabet, folded into soluble, stable and catalytically active structures, even though their stabilities and activities were not as high as those of the parent protein. Notably, many but not all of the reduced-set amino acids coincide with those plausibly abundant in primitive Earth. The inconsistent amino acids appeared to be important for catalytic activity but not for stability. Therefore, our findings suggest that the prebiotically abundant amino acids were used for creating stable protein structures and other amino acids with functional side chains were recruited to achieve efficient catalysis.

## Introduction

Terrestrial life uses nucleic acid polymers as the genetic molecules and, most often, proteins as the functional molecules. The nucleic acid polymers DNA and RNA contain the information for the amino acid sequences of proteins, and proteins are involved in the replication of the nucleic acid polymers. Therefore, regarding the origin of life, which emerged earlier, nucleic acids or proteins, had been a chicken and egg paradox. However, the findings of abiotic synthesis and polymerization of ribonucleotides^1,2^, replication of RNA by a ribozyme^3^, and a cross-chiral RNA polymerase ribozyme that synthesizes the RNA polymer in its own mirror image^4^ have solved the paradox; the RNA world most likely existed prior to the emergence of proteins. The next question involves the transition from the RNA world to the RNA-protein world. Therefore, unveiling the earliest protein synthesis system, which was the process to express the genetic information on RNA to produce proteins, is a key to understanding the origin and early evolution of life. However, whether the protein itself or the translation system emerged first remains a new chicken and egg paradox.

The size of the amino acid alphabet used in the earliest protein synthesis must be closely related to the origin and early evolution of the genetic code. Crick proposed the ‘frozen accident theory’ where development of the modern genetic code table was entirely a matter of chance^5^. In contrast, some theories have rationalized the evolution of the genetic code with different conclusions^6–12^. However, these theories commonly proposed that the earliest genetic code table involved much fewer than 20 amino acids and the modern genetic code table has progressively evolved from the primitive one by gradually incorporating new amino acids into the repertoire. Products of spark discharge experiments under conditions mimicking a plausible primitive environment and the amino acid composition in meteorites support the idea that only a subset of the genetically coded 20 amino acids were available in the prebiotic environment and used for the earliest protein synthesis^13–18^. Reasons why the 20 amino acids were selected have been also argued^19–22^.

Even if early proteins were composed of a limited set of amino acids, their structures should have been stable to the extent that they could express the biological functions. To date, several studies have shown that the full set of 20 genetically coded amino acids is not necessarily essential to produce a stable native-like protein structure and/or a catalytic function. A *de novo* designed four-helix bundle protein was synthesized with a reduced alphabet of seven amino acids^23^. The 93-residue, predominantly α-helical AroQ chorismate mutase was reconstructed from a 9-amino acid alphabet and was still catalytically active^24^. The addition of two more amino acid letters to the 9-amino acid enzyme dramatically improved its stability and activity^25^. However, it is still unclear whether the principles discerned from studies using simple helical proteins can be applied to topologically much more complex proteins. Although the catalytic activity associated with the more topologically complex, 213-residue *Escherichia coli* orotate phosphoribosyltransferase was achieved with a 13-amino acid alphabet, the 13-amino acid variant was substantially less stable than the wild-type protein^26^. Moreover, the reduced amino acid repertoires were arbitrarily chosen in those studies.

To reconstruct a simplified protein composed of a systematically chosen reduced amino acid set, an ancestral nucleoside diphosphate kinase (NDK) was used as the initial scaffold for restricting its amino acid usage to a reduced set while retaining a stable structure and catalytic activity. We previously resurrected several ancestral sequences of NDK that seem to represent ancestral proteins hosted by the last common ancestors of Archaea and of Bacteria^27,28^. In this study, one of the resurrected NDKs, named Arc1, was modified to simplify its amino acid usage. We used Arc1 because the ancestral NDK is extremely thermally stable, which is a plausible characteristic of primitive proteins^27,28^. The unfolding midpoint temperature of Arc1 is 114 °C (Supplementary Fig. S1)^27^. Its crystal structure has been solved to 2.4-Å resolution, showing that the protomers self-associate as a hexameric structure^27^. The arrangement of the protomers is quite similar to that of all known hexameric NDKs. Arc1 does not contain any cysteine residues and therefore consists of 19 amino acid types (Supplementary Table 1). By revealing the minimal set of amino acids that is essential for reconstructing a stable and active NDK, and then comparing the minimal amino acid set with amino acids that were plausibly available in the primitive Earth’s environment as proposed by earlier geochemical studies, we approximated the minimal amino acid composition of proteins that made primitive life possible.

## Results

### Effect of eliminating one amino acid letter on the stability and activity of Arc1

**Figure 1:**
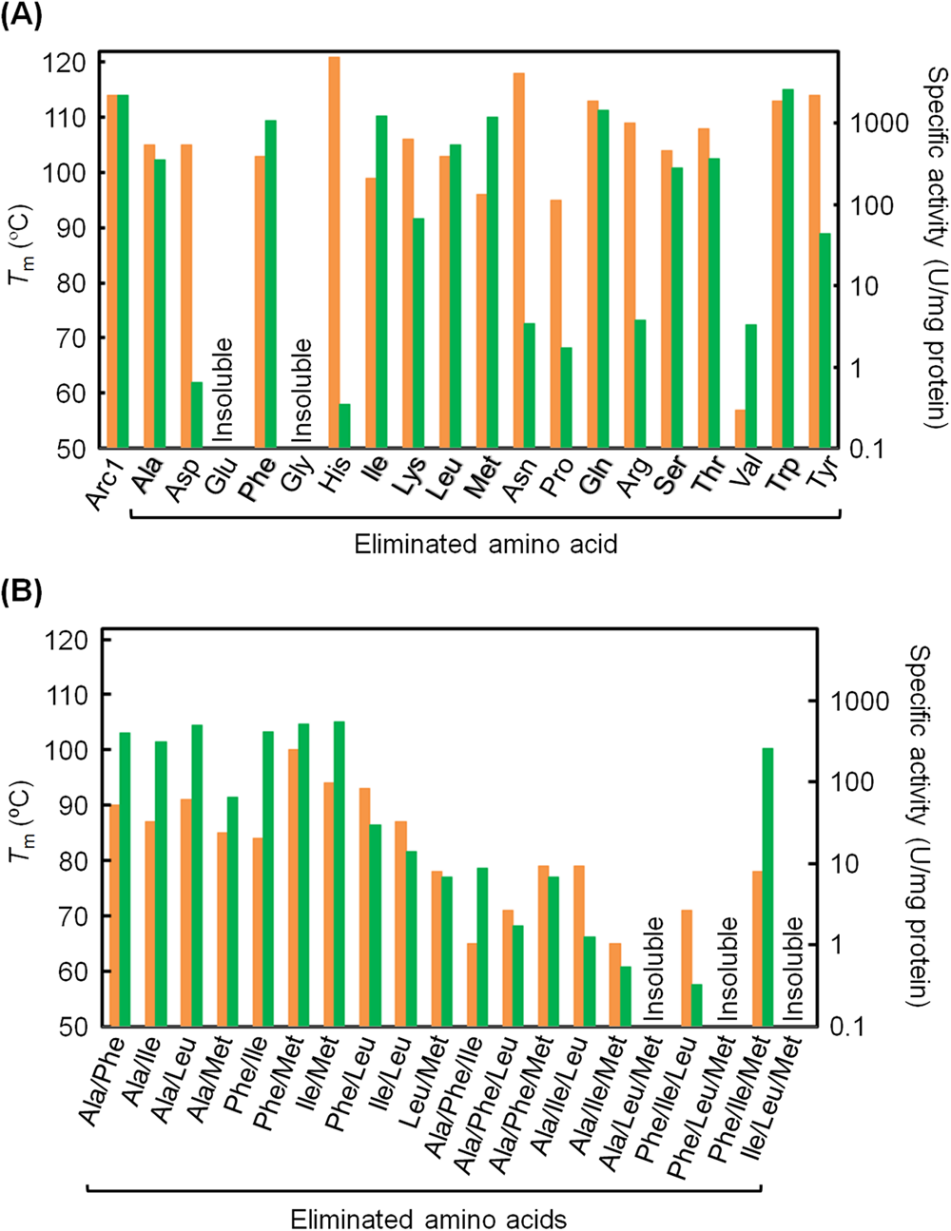
Thermal stabilities and specific activities of Arc1 and its simplified variants. (**A**) *T*_m_ (orange) and specific activities (green) for Arc1 and its simplified variants, each devoid of one amino acid letter. Specific activity of the variant devoid of valine was determined at 50 °C and those of all other proteins were at 70 °C. (**B**) *T*_m_ and specific activity at 70 °C for simplified variants devoid of multiple amino acid letters.

We constructed 19 simplified variants of Arc1, each of which is devoid of one amino acid letter. Because Arc1 is already devoid of cysteine, the variants are each comprised of an 18-amino acid alphabet. In each of those variants, one amino acid letter was eliminated by replacing that amino acid residue with the amino acid residue that is most frequently found at the corresponding position in the multiple amino acid sequence alignment of 309 extant NDK sequences. We expected that frequently occurring amino acids at a particular position had been favorably selected at that position during the course of NDK evolution. In addition, frequent amino acids often contribute to protein’s stability to greater extent than do less frequent amino acids^29^. Completely conserved residues were replaced by chemically similar amino acids. For constructing variants lacking methionine in this study, the N-terminal residue was not considered. Two variants that were devoid of cysteine and either glycine or glutamate appeared to be insoluble (Fig. 1A) and therefore could not be subjected to further analysis. It is likely that the presence of glycine and glutamate is crucial for the proper folding and/or thermodynamic stability of the protein.

To assess the thermal stabilities of the remaining 17 variants, each of which was comprised of an 18-amino acid alphabet, we carried out temperature-induced unfolding experiments on each protein by monitoring the changes in ellipticity at 222 nm as a function of temperature. For each protein, identical melting profiles were produced within experimental error in duplicate measurements. The thermal melting profiles of the simplified proteins showed a single transition, and the midpoint transition temperatures were used to compare their thermal stabilities. We also determined the specific activity of each simplified variant at 70 °C except in the case of the variant lacking valine, which was analyzed at 50 °C because the variant was not stable at 70 °C. NDK catalyzes the transfer of the γ-phosphate of a nucleoside triphosphate to a nucleoside diphosphate^30^. Figure 1A illustrates the thermal unfolding midpoint temperatures and the specific activities of Arc1 and its simplified variants, showing that elimination of some amino acid letters from the sequence of Arc1 exerts large effects on its stability and/or activity. In particular, valine is crucial for both stability and activity. Elimination of aspartate, histidine, asparagine or arginine did not affect the stability but substantially reduced the catalytic activity. Elimination of proline or tyrosine moderately reduced the activity. In contrast, the remaining ten amino acids (A, F, I, K, L, M, Q, S, T, W) could easily be eliminated from the sequence of Arc1 without compromising its structure and function. Thus, all of the amino acid letters do not contribute equally to the stability and activity of Arc1 and, in subsequent experiments, these ten amino acid letters were targeted to be eliminated to produce extensively simplified variants that were composed of significantly fewer than 20 amino acid letters.

### Construction of simplified Arc1 variants devoid of multiple-amino acid letters

**Figure 2:**
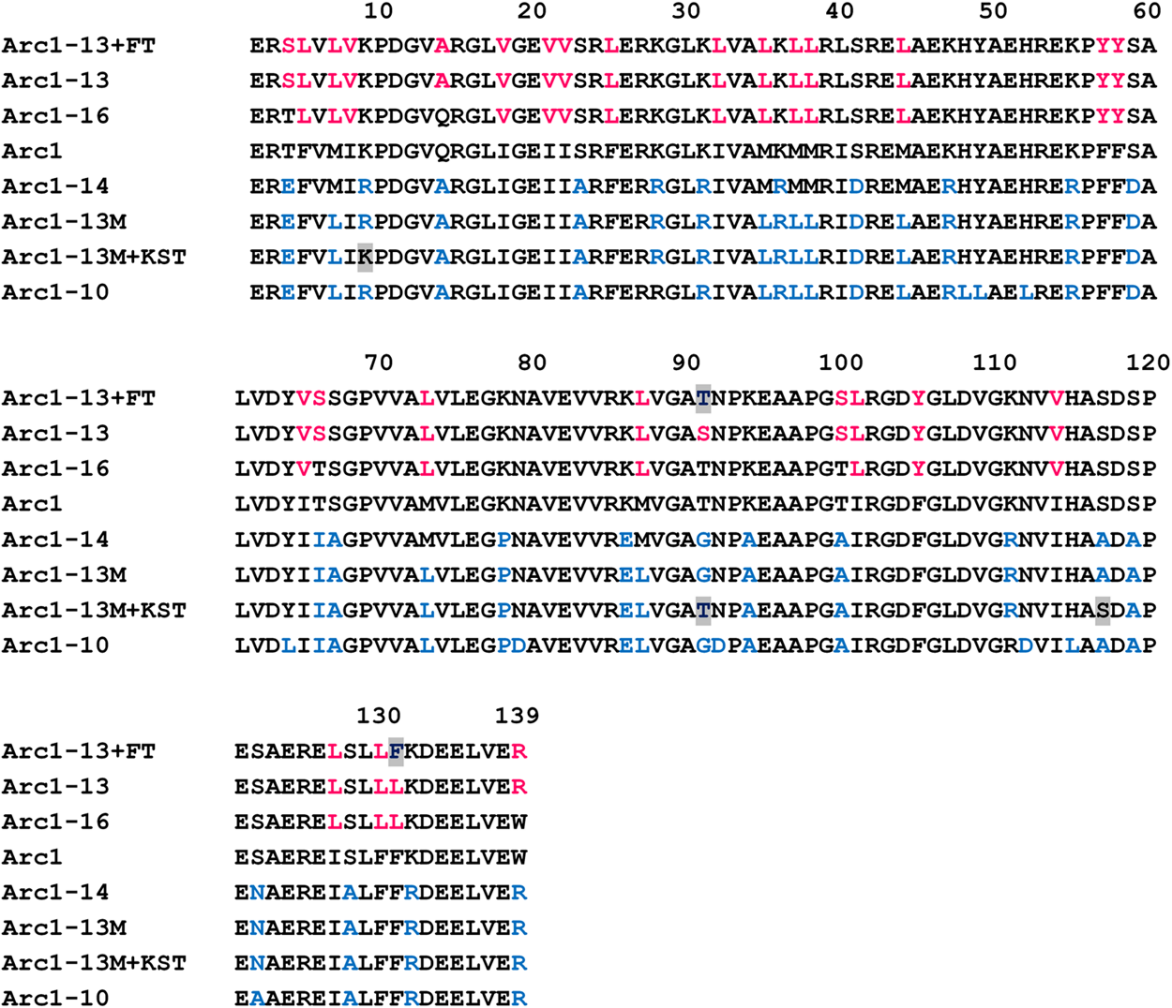
Amino acid sequences of Arc1 and its simplified variants. N-terminal residue(s) were omitted from this alignment. The substituting amino acids are shown in magenta or blue. Gray shading indicates residues restored to the original ones because they are almost completely conserved among extant NDK sequences.

We tested whether two or more non-polar amino acid letters could simultaneously be eliminated without substantial loss of stability and activity of Arc1. We targeted A, F, I, L and M for elimination. The non-polar amino acids do not contain any functional side chains and therefore may not be directly involved in NDK’s catalytic function. We excluded V from the analysis because elimination of valine substantially affected the stability and activity of Arc1 (Fig. 1A). Simultaneous elimination of any two of A, F, I and M by replacing other amino acids did not substantially affect the stability and activity, but elimination of L and one more non-polar letters reduced the catalytic activity (Fig. 1B). We also eliminated any three of A, F, I, L and M together. One of the resulting proteins, Arc1-16 (Fig. 2 and Supplementary Table S1), was reasonably thermally stable (*T*_m_ = 78 °C; Fig. 1B, Table 1 and Supplementary Fig. S1) and its specific activity was also significant (260 units/mg at 70 °C; Table 1). We further eliminated three amino acid letters (Q, T, W) from Arc1-16, thus producing Arc1-13 (Fig. 2 and Supplementary Table S1). The thermal stability of Arc1-13 (*T*_m_ = 74 °C) was substantially lower than that of Arc1 (*T*_m_ = 114 °C) but still much higher than that of an extant mesophilic NDK from *Bacillus subtilis* (*T*_m_ = 57 °C; Table 1 and Supplementary Fig. S1). The specific activity of Arc1-13 was 12 units/mg at 50 °C (Table 1). Therefore, a reduced alphabet, consisting only of 13 amino acid letters, is sufficient to achieve high thermal stability and catalytic function.

**Table 1:** Summary of the reconstructed NDK and its simplified variants as well as an extant mesophilic NDK.

	Eliminated amino acids	Oligomeric structure	*T*_m_ (°C)^a^	Specific activity (unit/mg)^b^
Arc1	C	Hexamer	114	2,100 (70 °C) 1,200 (50 °C)
Arc1-16	C, F, I, M	Hexamer	78	260 (70 °C)
Arc1-13	C, F, I, M, Q, T, W	(Hexamer)+dimer	74	12 (50 °C)
Arc1-13+FT	C, I, M, Q, W	Hexamer	81	160 (50 °C)
Arc1-14	C, K, Q, S, T, W	Dimer	81	8.6 (70 °C)
Arc1-13M	C, K, Q, S, T, W, M	Dimer	74	0.15 (50 °C)
Arc1-13M+KST	C, Q, W, M	Dimer	79	2.6 (50 °C)
Arc1-10	C, K, Q, S, T, W, M, H, N, Y	Dimer	85	<0.040 (50 °C)
*B. subtilis* NDK		Hexamer	57	860 (50 °C)

^a^*T*_m_ is the temperature corresponding to 50% denaturation as determined by monitoring the change in ellipticity at 222 nm (Fig. 2).

^b^Reaction temperatures are given in parentheses. The specific activities of Arc1, Arc1-16, and Arc1-14 were determined at 70 °C because the maximum activity was observed that temperature under the conditions used, but the activities of other proteins were determined at 50 °C to avoid the effect of protein denaturation.

To explore alternative reduced alphabets that can encode a stable and active NDK, we also eliminated the five amino acids, Lys, Gln, Ser, Thr and Trp, at one time from the sequence of Arc1 by replacing them with other amino acids. The five amino acids could easily be eliminated from Arc1 without significantly affecting its stability or activity (Fig. 1A). The resulting Arc1-14 consisted of a 14-amino acid alphabet (Fig. 2 and Supplementary Table S1). Temperature-induced unfolding and catalytic activity measurements revealed that Arc1-14 showed a 33 °C lower unfolding midpoint temperature (81 °C; Table 1 and Supplementary Fig. S1) and a 244-fold lower specific activity at 70 °C (8.6 units/mg) compared to those of Arc1 (Table 1). Thus, Arc1-14 is also a stable and active protein, even though its stability and activity were not as high as those of Arc1. Therefore, catalytic function associated with a stable protein structure may be achieved with several subsets of the 20 coded amino acids.

### Oligomeric structures as studied by analytical gel filtration

The quaternary structures for the original Arc1 and its simplified variants were investigated by analytical gel filtration using Superdex 200 resin with initial protein concentrations of 20 µM. We previously showed that protomers of Arc1 self-associate into a hexameric structure, similar to the quaternary structure of many extant NDKs^31–33^, and the manner of the hexameric assembly of Arc1 is identical to those of all known extant hexameric NDKs^34^. The elution profile shows that Arc1-16 migrated as a single molecular species with a retention volume corresponding to the molar mass of a hexamer (Table 1). In contrast, Arc1-13 exists predominantly as a dimer, but a small elution peak corresponding to the hexameric state was also observed (Table 1). Arc1-14 also eluted as a single peak but its elution volume corresponds to a dimeric structure (Table 1). Because Arc1-14 and Arc1-13 form stable structures that exhibit catalytic activity, hexamerization is not necessarily essential for proper folding of the protomer and catalytic function of the enzyme.

### Further exclusion of amino acid letters from the simplified Arc1 variants

Despite the significant simplification of the amino acid sequences, Arc1-13 and Arc1-14 still exhibited significant stability and a detectable level of catalytic activity. In order to simplify these variants further, we eliminated A, K, S, or Y from the sequence of Arc1-13, and A, F, I, L, M, or Y from the sequence of Arc1-14, thus yielding Arc1-12A/K/S/Y and Arc1-13A/F/I/L/M/Y (Supplementary Fig. S2) because those amino acid letters could be eliminated from the sequence of Arc1 without compromising its stability or catalytic activity (Fig. 1A). The solubility of Arc1-12A appeared to be low, which caused the variant to precipitate when purification was attempted. The other variant was successfully purified and the far-UV circular dichroism (CD) spectra, except for that of Arc1-12S, were indicative of significant secondary structure. The changes in ellipticity as a function of temperature for Arc1-12K, Arc1-12Y, Arc1-13I, Arc1-13L, Arc1-13M, and Arc1-13Y showed cooperative two-state transitions (Supplementary Fig. S3). In contrast, atypical unfolding curves were obtained for Arc1-12S, Arc1-13A, and Arc1-13F, suggesting that these variants did not fold properly. The measurements of γ-phosphate transferring activity showed that only Arc1-13M exhibited a detectable level of catalytic activity although its specific activity at 50 °C (0.15 units/mg) was 8,000 times lower than that of Arc1 (Table 1). The stability (*T*_m_ = 74 °C) and activity of Arc1-13M, together with those of Arc1-13, demonstrated that sets of 13 amino acid types are sufficient to produce a stable and active NDK.

## Discussion

It is hard to believe that the modern protein synthesis system involving the full set of 20 amino acids was also used in the earliest life. Rather, it is more plausible that an earlier system for protein synthesis was much simpler and involved only a limited number of amino acid letters, and that the protein synthesis system has progressively evolved from the earliest one by gradually recruiting new amino acids into the set of protein-coding amino acids. More than 50 years ago, Eck and Dayhoff hypothesized the origin and early evolution of ferredoxin^35^. They proposed that a primitive form of the protein emerged by duplicating a shorter protein that might have been composed of an eight-amino acid alphabet. Because they traced the evolution of ferredoxin back without the currently available sophisticated computer programs for ancestral sequence reconstruction, their assertion was highly speculative. However, the concept illustrated by Eck and Dayhoff was later embodied as ribosomal RNA-based phylogenies^36,37^ and computer-assisted ancestral sequence reconstructions^38,39^. Currently, the characteristics of ancient organisms and the environments of their biospheres can be estimated by characterizing ancestral proteins reconstructed using expanded genome data available in public databases and advanced phylogenetic analysis techniques^27,40–43^. The present study also relies on the reconstruction technique because the previously reconstructed ancestral NDK, Arc1, was used as the template for restricting the building units to a subset of genetically coded amino acids.

**Figure 3:**
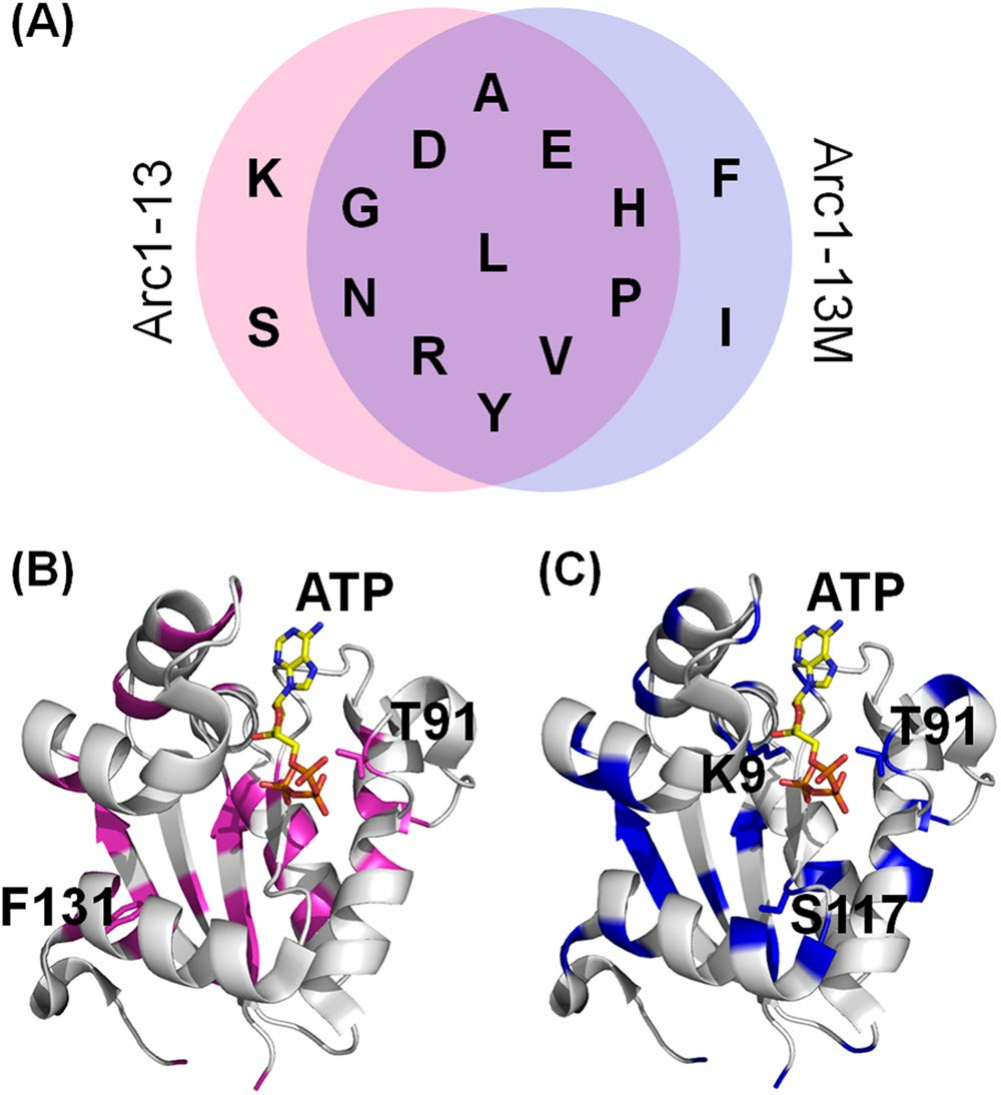
Two simplified proteins composed of 13 amino acid letters. (**A**) Venn diagram comparing the amino acid letters included in the two simplified proteins. (**B**, **C**) The monomer structures of Arc1 (PDB code, 3vvt) with ATP. The bound ATP was modeled by superimposing the Arc1 structure with the structure of *Thermus thermophilus* NDK with bound ATP (PDB code, 1wkl). (**B**) Amino acid residues replaced by a reduced-set of 13 amino acids in Arc1-13 are shown as a magenta backbone ribbon. The residues at positions 91 and 131, which are highly conserved among extant NDKs, are shown as sticks models. (**C**) Amino acid residues replaced with a reduced-set of 13 amino acids in Arc1-13M are rendered in blue. The residues at positions 9, 91, and 117 are shown in sticks models.

As reported herein, we obtained two simplified Arc1 variants (i.e. Arc1-13 and Arc1-13M), both of which showed reasonably high thermal stability and a detectable level of catalytic activity albeit being comprised of a 13-amino acid alphabet. As shown in Fig. 3, eleven amino acid letters are commonly included in the sequences of the two simplified proteins. Therefore, we refer these eleven amino acids to as the ‘essential amino acids’. We would like to note that the original fraction of essential residues in the ancestral protein Arc1 is not significantly higher than those in modern NDKs. What can we learn from the eleven essential amino acids? It is reasonable to predict that the earliest protein was synthesized with amino acids that could be obtained from the prebiotic Earth’s environment. Miller simulated primitive Earth’s possible environments and demonstrated that ten (A, D, E, G, I, L, P, S, T, V) of the 20 coded amino acids were synthesized in abiotic environments (Supplementary Table S2)^13,18^. Eight (A, D, E, G, I, L, P, V) of Miller’s amino acids were also found in the Murchison meteorite (Supplementary Table S2)^17,19^. Organic compounds, such as amino acids found in meteorites might have originated from the universe^44^. Assuming that the composition of amino acids in the Murchison meteorite reflects the abundance of amino acids in prebiotic environments, these eight amino acids are thought to have existed abundantly in primitive Earth. Primitive proteins must have been synthesized by utilizing amino acids present in the environment before the invention of amino acid biosynthetic pathways^6^. Therefore, the eight amino acids found in both the products of the Miller’s experiments and the Murchison meteorite were plausibly involved in the earliest protein synthesis. All eight of these amino acids, except for isoleucine, were found to be essential amino acids for stable and active NDKs (Supplementary Table S2). Another group reported that simplified β-trefoil proteins whose amino acid usages were biased toward the ten Miller’s amino acids were foldable in a halophilic environment^45^. Therefore, reconstruction of stable and active proteins composed of a limited amino acid set provides additional support for the abundantly present amino acids in the paleoenvironment that has been inferred from geochemical studies.

However, four (H, N, R, Y) of the essential amino acids are not found in both Miller’s experiment and the Murchison meteorite (Supplementary Table S2). Therefore, it has been thought that they were rarely synthesized in prebiotic environments^19^. Our interpretation for this disagreement is that primitive proteins only served as environments for chemical reactions and were not directly involved in catalysis. It has been pointed out that, during the early stage of evolution, amino acids were not selected for their ability to promote catalytic reactions, but for allowing the formation of stable and soluble tertiary structures^46^. This idea supposes that other molecules such as RNA, cofactors and metals might have played a central role in catalytic function. Indeed, most of the amino acids that plausibly existed in prebiotic environments do not contain functional side chains that are important for catalysis. In accordance with this idea, histidine, asparagine, arginine and tyrosine are essential only for catalytic activity and not for conformational stability (Fig. 1A). Arc1-10, which was produced by eliminating histidine, asparagine and tyrosine from Arc1-13M (Fig. 2 and Supplementary Table S1), recovered the thermal stability by 11 °C (Table 1 and Supplementary Fig. S4), further supporting this idea. Therefore, these amino acids were plausibly recruited into the protein synthesis system later than the eight amino acid letters, promoting a diversified catalytic repertoire by providing new functional groups. However, the possibility that the four amino acid letters (H, N, R, Y) were abiotically synthesized in some way cannot be ruled out. Sutherland and colleagues recently reported abiotic synthesis pathways for precursors to arginine and asparagine (Supplementary Table S2)^47^. Prebiotic syntheses of histidine and imidazolide have also been proposed^47–50^.

Primordial proteins might have been comprised of a reduced set of amino acids and subsequent addition of new amino acids would expand the geometrical and functional diversities of side chains, thus plausibly improving the stability and activity of proteins. Among the 309 extant NDK sequences, lysine, threonine, serine and phenylalanine are almost completely conserved at positions 9, 91, 117, and 131, respectively. Therefore, we restored the residues found at positions 91 and 131 of Arc1-13 to the original amino acids, threonine and phenylalanine, respectively, and the residues at positions 9, 91 and 117 of Arc1-13M to the original amino acids, lysine, threonine and serine, respectively (Fig. 2). The resulting proteins, Arc1-13 + FT and Arc1-13M + KST largely recovered both stability and catalytic activity (Table 1 and Supplementary Fig. S4). Accordingly, similar to a previous report by Hilvert and colleagues^25^, our results demonstrate that the structure and function of the proteins composed of a reduced set of amino acids could be improved by incremental addition of new amino acids. If similar improvements occurred for primitive proteins, expansion of the amino acid repertoire would have improved the fitness of the host organisms and thus may have driven the evolution of early life.

## Materials and Methods

### Protein preparation

The sequences of the genes encoding the simplified proteins were determined by reverse translation of the simplified Arc1 sequences so that the codon usage was optimized for an *Escherichia coli* expression system. The genes, cloned into pTAKN-2, were synthesized by Eurofins Genomics (Tokyo, Japan). The genes encoding the simplified proteins were excised from the pTAKN-2 constructs by digestion with *Nde*I and *Bam*HI (New England Biolabs Japan, Tokyo) and then subcloned into pET23a(+) (Merck, Tokyo). The resulting expression plasmids were used to transform *E. coli* Rosetta2 (DE3) (Merck, Tokyo) and the transformants were cultivated in Luria-Bertani medium supplemented with 150 μg/mL ampicillin at 37 °C. Gene expression was induced using Overnight Express Autoinduction system 1 reagents (Merck, Tokyo). *E. coli* cells were then harvested, disrupted by sonication, and heat-treated at 60 or 70 °C for 20 min to precipitate *E. coli* proteins. After centrifugation at 15,000 × *g* at 4 °C for 30 min, the simplified proteins were purified from the supernatants by successive column chromatography through HiTrap Q and Resource Q (GE Healthcare Japan, Tokyo).

Preparation of Arc1-13, Arc1-12A, Arc1-12K, Arc1-12S, and Arc1-12Y was performed so that the N-terminal methionine was also eliminated. Each gene was PCR amplified from the pTAKN-2 construct using KOD-plus DNA polymerase (Toyobo, Osaka) and a pair of primers. The downstream primers contained a *Hin*dIII restriction site at its 5′ terminus. The PCR products were then digested with *Hin*dIII (New England Biolabs Japan, Tokyo) and subcloned into the *Stu*I-*Hin*dIII site of pQE30Xa (Qiagen, Tokyo) so that the simplified proteins would be N-terminally His-tagged. For protein production, *E. coli* M15 (pREP4) strain was transformed with the resulting expression plasmid and then cultivated in Luria-Bertani medium supplemented with ampicillin (150 µg/ml). Expression was induced with 0.1 mM isopropyl β-D-1-thiogalactopyranoside. After overnight cultivation, cells were harvested by centrifugation and disrupted by sonication. Precipitate was removed by centrifugation at 15,000 × *g* and 4 °C for 30 min and soluble proteins were purified using a HisTrap FF nickel affinity column (GE Healthcare Japan, Tokyo). The N-terminal His-tag (excepting residues G_−6_G_−5_G_−4_G_−3_G_−2_A_−1_ or G_−6_G_−5_G_−4_G_−3_G_−2_L_−1_) was then removed by digestion with Factor Xa protease (Qiagen, Tokyo). The released His-tag and Factor Xa protease were removed from the solution by passage through a HisTrap FF nickel affinity column and a HiTrapQ anion exchange column (GE Healthcare Japan, Tokyo). The purified proteins thus did not contain methionine at their N-terminus; instead they contained an N-terminal 6-residue extension (GGGGGA or GGGGGL). The same method could not be applied to the preparation of Arc1-13M because the variant was degraded during proteolysis with Factor Xa.

The purity of each protein was >95% as judged by the results of SDS-polyacrylamide gel electrophoresis followed by Coomassie Blue staining.

### Analytical methods

Protein concentrations were determined by measuring the OD_280_ of the protein solutions according to the procedure reported by Pace and colleagues^51^, who improved the procedure of Gill and von Hippel^52^. Thermal unfolding curves were obtained using a J-720 spectropolarimeter (Jasco, Hachioji) equipped with a programmable temperature controller and a pressure-proof cell compartment that prevented the solutions from bubbling and evaporating at high temperatures. The path-length of the cell used was 0.1 cm. Proteins were diluted to 25 µM with 20 mM potassium phosphate (pH 6.0), 50 mM KCl, 1 mM EDTA. Temperature was increased at a rate of 1.0 °C/min.

Enzymatic activity was determined from the results of an assay where the increase in the amount of ATP, a product of the reaction, was measured using a luminescent kinase assay kit, Kinase-Glo (Promega), as described previously^27^. The kinase assay buffer was 50 mM HEPES (pH 8.0), 25 mM KCl, 10 mM (NH_4_)_2_SO_4_, 2.0 mM (CH_3_COO)_2_Mg, 1.0 mM DTT, 1.0 mM ADP and 2.5 mM GTP.

Oligomeric structures of the simplified proteins were determined by analytical gel filtration using Superdex 200 resin (column dimensions 1.0 × 30 cm; GE Healthcare Japan, Tokyo) equilibrated with 20 mM potassium phosphate (pH 6.0), 150 mM KCl, 1 mM EDTA. Protein, in an initial volume of 0.2 ml, was loaded onto the column at a flow rate of 0.5 ml/min. Apparent molar masses were determined from the elution volumes and a calibration curve produced using proteins of known molar masses and elution volumes.

## Electronic supplementary material

Supplementary Information
