# Supplementary material for: Comprehensive reduction of amino acid set in a protein suggests the importance of prebiotic amino acids for stable proteins
Source: Sci Rep. 2018 Jan 19;8:1227. doi: 10.1038/s41598-018-19561-1 (PMC5775292; doi:10.1038/s41598-018-19561-1)
Supplement: Supplementary file 1 — Supplementary Information [file 41598_2018_19561_MOESM1_ESM.pdf]

Comprehensive reduction of amino acid set in a protein suggests the importance of prebiotic amino acids for stable proteins

Rei Shibue[1], Takahiro Sasamoto[2], Masami Shimada[2], Zhang Bowen[1], Akihiko Yamagishi[2], Satoshi Akanuma[*,1]

[1]Faculty of Human Sciences, Waseda University, 2-579-15 Mikajima, Tokorozawa, Saitama 359-1192, Japan

[2]Department of Applied Life Science, Tokyo University of Pharmacy and Life Sciences, 1432-1 Horinouchi, Hachioji, Tokyo 192-0392, Japan

[*]**Corresponding author:** Satoshi Akanuma, E-mail: akanuma@waseda.jp

Supplementary Table S1. Amino acid compositions of Arc1 and its simplified variants constructed in this study.

Amino acid	Arc1	Arc1-16	Arc1-13	Arc1-13 +FT	Arc1-14	Arc1-13M	Arc1-13M +KST	Arc1-10
Ala	11	11	12	12	19	19	18	20
Cys	0	0	0	0	0	0	0	0
Asp	6	6	6	6	8	8	8	11
Glu	16	16	16	16	18	18	18	18
Phe	7	0	0	1	7	7	7	7
Gly	11	11	11	11	12	12	11	12
His	3	3	3	3	3	3	3	0
Ile	10	0	0	0	11	11	11	11
Lys	11	11	11	11	0	0	1	0
Leu	7	22	22	21	7	14	14	19
Met[a]	7	0	0	0	7	0	0	0
Asn	3	3	3	3	4	4	4	0
Pro	6	6	6	6	7	7	7	7
Gln	1	1	0	0	0	0	0	0
Arg	10	10	11	11	19	19	18	19
Ser	8	8	12	11	0	0	1	0
Thr	4	4	0	1	0	0	1	0
Val	14	20	20	20	14	14	14	14
Trp	1	1	0	0	0	0	0	0
Tyr	2	5	5	5	2	2	2	0

[a]The N-terminal methionine residues are not taken into account.

Supplementary Table S2. Comparison of the essential amino acids defined in this study with amino acids that are proposed to have been abundant in primitive Earth.

	Ala	Cys	Asp	Glu	Phe	Gly	His	Ile	Lys	Leu
Essential for NDK	+	–	+	+	–	+	+	–	–	+
Miller's experiment[1,2]	+	–	+	+	–	+	–	+	–	+
Murchison meteorite[3,4]	+	–	+	+	–	+	–	+	–	+
Sutherland's abiotic synthesis[5]	+	–	+	+	–	+	–	–	–	+

	Met	Asn	Pro	Gln	Arg	Ser	Thr	Val	Trp	Tyr
Essential for NDK	–	+	+	–	+	–	–	+	–	+
Miller's experiment	–	–	+	–	–	+	+	+	–	–
Murchison meteorite	–	–	+	–	–	–	–	+	–	–
Sutherland's abiotic synthesis	–	+	+	+	+	+	+	+	–	–

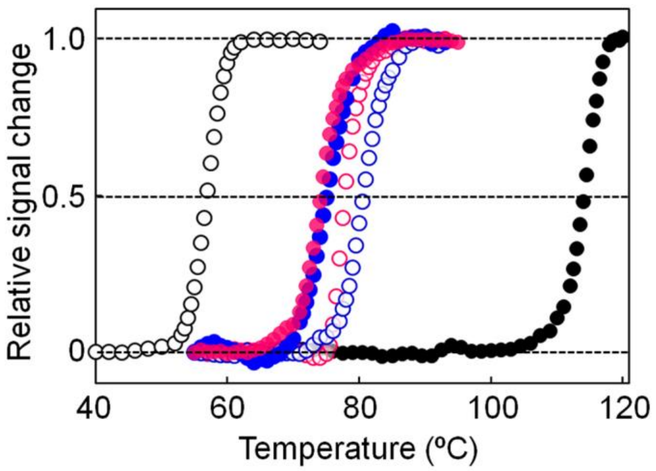

Supplementary Figure S1. Thermal unfolding monitored at an ellipticity at 222 nm. Each protein (25 μM) was dissolved in 20 mM potassium phosphate, pH 6.0, 50 mM KCl, 1 mM EDTA. The scan rate was 1.0°C/min. Identical unfolding curves were produced within experimental error for duplicate measurements. The plots were normalized with respect to the baselines of the native and denatured states. Black filled circles, Arc1; magenta open circles, Arc1-16; magenta filled circles, Arc1-13; blues open circles, Arc1-14; blue filled circles, Arc1-13M; black open circles, *B. subtilis* NDK.

```
                        10        20        30        40        50        60
Arc1-12A     ERSLVLVKPDGVRRGLVGEVVSRLERKGLKLVGLKLLRLSRELLEKHYEEHREKPYYSE
Arc1-12K     ERSLVLVRPDGVARGLVGEVVSRLERRGLRLVALRLLRLSRELAERHYAEHRERPYYSA
Arc1-12S     ERELVLVKPDGVARGLVGEVVARLERKGLKLVALKLLRLDRELAEKHYAEHREKPYYDA
Arc1-12Y     ERSLVLVKPDGVARGLVGEVVSRLERKGLKLVALKLLRLSRELAEKHHAEHREKPLLSA
Arc1         ERTFVMIKPDGVQRGLIGEIISRFERKGLKIVAMKMMRISREMAEKHYAEHREKPFFSA
Arc1-13A     EREFVMIRPDGVRRGLIGEIIGRFERRGLRIVGMRMMRIDREMLERHYEEHRERPFFDE
Arc1-13F     ERELVMIRPDGVARGLIGEIIARLERRGLRIVAMRMMRIDREMAERHYAEHRERPYYDA
Arc1-13I     EREFVMVRPDGVARGLVGEVVARFERRGLRLVAMRMMRLDREMAERHYAEHRERPFFDA
Arc1-13L     EREFVMIRPDGVARGVIGEIIARFERRGFRIVAMRMMRIDREMAERHYAEHRERPFFDA
Arc1-13M     EREFVLIRPDGVARGLIGEIIARFERRGLRIVALRLLRIDRELAERHYAEHRERPFFDA
Arc1-13Y     EREFVMIRPDGVARGLIGEIIARFERRGLRIVAMRMMRIDREMAERHHAEHRERPFFDA

                      70        80        90        100       110       120
Arc1-12A     LVDYVSSGPVVVLVLEGKNVVEVVRKLVGKSNPKESEPGSLRGDYGLDVGKNVVHGSDSP
Arc1-12K     LVDYVSSGPVVALVLEGPNAVEVVRSLVGASNPAEAAPGSLRGDYGLDVGRNVVHASDSP
Arc1-12S     LVDYVLAGPVVALVLEGKNAVEVVRKLVGAGNPKEAAPGALRGDYGLDVGKNVVHAADAP
Arc1-12Y     LVDLVSSGPVVALVLEGKNAVEVVRKLVGASNPKEAAPGSLRGDLGLDVGKNVVHASDSP
Arc1         LVDYITSGPVVAMVLEGKNAVEVVRKMVGATNPKEAAPGTIRGDFGLDVGKNVIHASDSP
Arc1-13A     LVDYIIGGPVVVMVLEGPNVVEVVREMVGPGNPLEREPGGIRGDFGLDVGRNVIHGGDGP
Arc1-13F     LVDYIIAGPVVAMVLEGPNAVEVVREMVGAGNPAEAAPGAIRGDYGLDVGRNVIHAADAP
Arc1-13I     LVDYMMAGPVVAMVLEGPNAVEVVREMVGAGNPAEAAPGALRGDFGLDVGRNVVHAADAP
Arc1-13L     MVDYIIAGPVVAMVVEGPNAVEVVREMVGAGNPAEAAPGAIRGDFGIDVGRNVIHAADAP
Arc1-13M     LVDYIIAGPVVALVLEGPNAVEVVRELVGAGNPAEAAPGAIRGDFGLDVGRNVIHAADAP
Arc1-13Y     LVDFIIAGPVVAMVLEGPNAVEVVREMVGAGNPAEAAPGAIRGDFGLDVGRNVIHAADAP

                     130      139
Arc1-12A     ESGERELSLLLKDEELVER
Arc1-12K     ESAERELSLLLRDEELVER
Arc1-12S     EAAERELALLLKDEELVER
Arc1-12Y     ESAERELSLLLKDEELVER
Arc1         ESAEREISLFFKDEELVEW
Arc1-13A     ENGEREIGLFFRDEELVER
Arc1-13F     ENAEREIALLLRDEELVER
Arc1-13I     ENAERELALFFRDEELVER
Arc1-13L     ENAEREIAYFFRDEEIVER
Arc1-13M     ENAEREIALFFRDEELVER
Arc1-13Y     ENAEREIALFFRDEELVER
```

Supplementary Figure S2. Amino acid sequences of Arc1 and its simplified variants devoid of seven or eight amino acid letters. N-terminal residues were omitted from this alignment. The adopted amino acids are shown in magenta or blue.

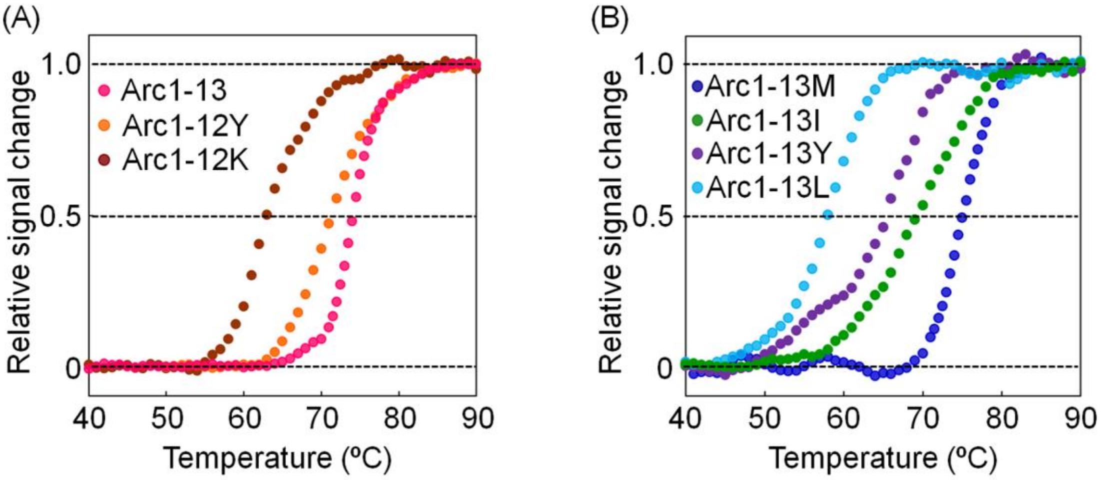

Supplementary Figure S3. Unfolding of simplified variants monitored at θ_{222}. Each enzyme (25 μM) was dissolved in 20 mM potassium phosphate, pH 6.0, 50 mM KCl, 1 mM EDTA. The scan rate was 1.0°C/min. Identical melting curves were obtained within experimental error for duplicate measurements. The plots were normalized with respect to the baselines of the native and denatured states.

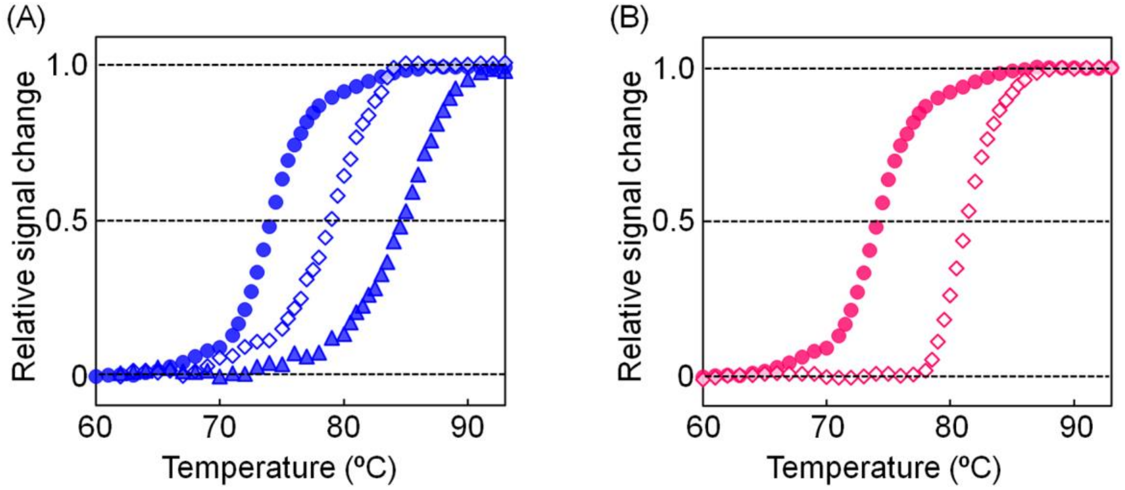

Supplementary Figure S4. Unfolding of simplified variants monitored at θ_{222}. Each enzyme (25 μM) was dissolved in 20 mM potassium phosphate, pH 6.0, 50 mM KCl, 1 mM EDTA. The scan rate was 1.0°C/min. Identical melting curves were obtained within experimental error for duplicate measurements. The plots were normalized with respect to the baselines of the native and denatured states. (A) Blue filled circles, Arc1-13M; blue open diamonds, Arc1-13M+KST; blue filled triangles, Arc1-10; (B) magenta filled circles, Arc1-13; magenta open diamonds, Arc1-13+FT.

Supplementary References

(1) Miller, S. L. A production of amino acids under possible primitive earth conditions. *Science* **117**, 528–529 (1953).

(2) Johnson, A. P. *et al.* The Miller volcanic spark discharge experiment. *Science* **322**, 404, (2008).

(3) Cronin, J. R. & Pizzarello, S. Amino acids in meteorites. *Adv Space Res* **3**, 5–18 (1983).

(4) Cleaves, H. J. 2nd. The origin of the biologically coded amino acids. *J Theor Biol* **263**, 490–498 (2010).

(5) Patel, B. H., Percivalle, C., Ritson, D. J., Duffy, C. D. & Sutherland, J. D. Common origins of RNA, protein and lipid precursors in a cyanosulfidic protometabolism. *Nat Chem* **7**, 301–307 (2015).
